# Restoring Mitochondrial Function and Muscle Satellite Cell Signaling: Remedies against Age-Related Sarcopenia

**DOI:** 10.3390/biom14040415

**Published:** 2024-03-28

**Authors:** Emanuele Marzetti, Biliana Lozanoska-Ochser, Riccardo Calvani, Francesco Landi, Hélio José Coelho-Júnior, Anna Picca

**Affiliations:** 1 Fondazione Policlinico Universitario “Agostino Gemelli” IRCCS, L.go A. Gemelli 8, 00168 Rome, Italy; riccardo.calvani@unicatt.it (R.C.); francesco.landi@unicatt.it (F.L.); 2 Department of Geriatrics, Orthopedics and Rheumatology, Università Cattolica del Sacro Cuore, L.go F. Vito 1, 00168 Rome, Italy; coelhojunior@hotmail.com.br; 3 Department of Medicine and Surgery, LUM University, 70010 Casamassima, Italy; lozanoska-ochser@lum.it; 4 DAHFMO Unit of Histology and Medical Embryology, Sapienza Università di Roma, P.le Aldo Moro 5, 00185 Rome, Italy

**Keywords:** aging, cytokines, inflammation, mitochondrial dysfunction, mitochondrial-derived vesicles, muscle fibrosis, muscle injury, muscle wasting, muscle satellite cells, skeletal muscle fibers

## Abstract

Sarcopenia has a complex pathophysiology that encompasses metabolic dysregulation and muscle ultrastructural changes. Among the drivers of intracellular and ultrastructural changes of muscle fibers in sarcopenia, mitochondria and their quality control pathways play relevant roles. Mononucleated muscle stem cells/satellite cells (MSCs) have been attributed a critical role in muscle repair after an injury. The involvement of mitochondria in supporting MSC-directed muscle repair is unclear. There is evidence that a reduction in mitochondrial biogenesis blunts muscle repair, thus indicating that the delivery of functional mitochondria to injured muscles can be harnessed to limit muscle fibrosis and enhance restoration of muscle function. Injection of autologous respiration-competent mitochondria from uninjured sites to damaged tissue has been shown to reduce infarct size and enhance cell survival in preclinical models of ischemia–reperfusion. Furthermore, the incorporation of donor mitochondria into MSCs enhances lung and cardiac tissue repair. This strategy has also been tested for regeneration purposes in traumatic muscle injuries. Indeed, the systemic delivery of mitochondria promotes muscle regeneration and restores muscle mass and function while reducing fibrosis during recovery after an injury. In this review, we discuss the contribution of altered MSC function to sarcopenia and illustrate the prospect of harnessing mitochondrial delivery and restoration of MSCs as a therapeutic strategy against age-related sarcopenia.

## 1. Introduction

Sarcopenia is a neuromuscular disease that involves dynapenia and muscle atrophy with loss in physical performance used to assess its severity [1]. This condition predisposes to an increased risk of negative health-related outcomes and has an impact on the prognosis of acute and chronic diseases [2,3,4]. Sarcopenia has a complex pathophysiology that involves, among other factors, metabolic dysregulation and detrimental changes in muscle ultrastructure. The latter phenomenon includes a reduction in the size and number of muscle fibers, motor neurons, and satellite cells, as well as the transition from fast to slow myosin isoforms (i.e., type II to type I fiber switch) [5,6,7]. At the clinical level, these changes translate into a progressive decline in skeletal muscle mass, strength, and function that eventually culminate into negative health-related events (e.g., disability) [8].

Declines in mitochondrial quality and quantity have been causally linked with skeletal myocyte dyshomeostasis and have been included among the factors contributing to age-related sarcopenia [9]. Other intracellular systems have also been implicated in the regulation of muscle homeostasis, including the balance between muscle protein synthesis via the mechanistic target of rapamycin (mTOR) and degradation through the AMP-activated protein kinase (AMPK)-driven inhibition of mTOR [10]. However, the connections among mitochondrial activities and mTOR/AMPK signaling during age-related sarcopenia have only partially been deciphered.

Although most studies on sarcopenia have focused on intrinsic changes and signaling pathways of skeletal muscle, other factors, including the coordinated activity of resident immune and non-immune cells of the muscle microenvironment, are critical for muscle health, repair, and regeneration [11]. In such a view, trained immunity, or inflammatory memory, for which the exposure of innate immune cells to inflammatory factors induces epigenetic reprogramming, has taken the stage from a muscle-centric view and envisions an immunological-assisted muscle wasting. However, these aspects have only partially been investigated in aging and sarcopenia, and they have not been explored as possible targets for interventions.

Among the drivers of intracellular and ultrastructural changes of muscle fibers in sarcopenia, mitochondria and their trafficking have been shown to play relevant roles [12]. Furthermore, mononucleated muscle stem cells/satellite cells (MSCs) have been attributed to critical functions in muscle repair after injury [13,14]. The involvement of mitochondria in supporting MSC-directed muscle repair is less understood. However, there is evidence that a reduction in mitochondrial biogenesis blunts muscle repair [15]. This suggests that the replenishment of functional mitochondria in injured muscles may reduce muscle fibrosis and assist in rescuing muscle function.

Pioneering studies have tested mitochondrial transplant as a strategy for the recovery of tissue homeostasis in different settings [16,17]. For instance, the injection of autologous respiration-competent mitochondria during ischemia–reperfusion injury from uninjured sites to damaged tissue was able to reduce infarct size and enhance cell survival in rabbits [18]. These findings have been confirmed in subsequent larger preclinical investigations [16,19] and finally reached the clinical stage [20]. Donor mitochondria can also be incorporated into MSCs to ameliorate lung and cardiac tissue repair [21,22]. This strategy has also been tested for regeneration purposes in traumatic injured muscle [23]. Recently, the systemic delivery of donor mitochondria has been shown to enhance muscle regeneration and restore muscle mass and function while reducing fibrosis during recovery from an injury [23]. Whether this strategy could serve as a remedy against age-related sarcopenia remains to be established, although this possibility holds promise as a therapeutic strategy. In this review, we discuss ultrastructural and biological changes that occur with aging in the skeletal muscle, with a focus on MSCs. We also illustrate the possibility of harnessing mitochondrial delivery and restoration of MSCs as a remedy against sarcopenia.

## 2. Ultrastructural Changes in the Aged Skeletal Muscle

**Figure 1:**
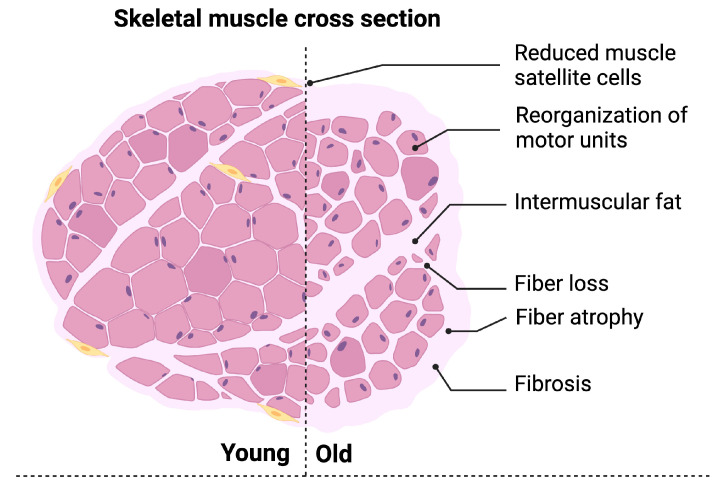
Schematic representation of muscle ultrastructural changes associated with aging. Notable changes include a reduced number and function of muscle satellite cells, reorganization of motor units, fiber loss and/or atrophy, and increased fibrosis. Created with https://www.biorender.com/ (accessed on 16 February 2024).

Multiple ultrastructural changes have been described in the skeletal muscle of aged animal models and humans (Figure 1).

One remarkable intrinsic change that accompanies aging is the reorganization of motor units that precedes muscle fiber type re-grouping and atrophy [24]. A spatial organization of motor units and fiber types has been observed in preclinical models. In particular, the tibialis anterior muscle of young mice shows the generation of a surface-to-depth gradient of muscle fibers, with the largest units and fibers expressing type IIb myosin heavy chain (MyHC) localized at the muscle surface and small motor units with type IIa MyHC located in the deeper region of the muscle. Among these, motor unit types with intermediate size and fibers expressing IIx MyHC isoforms have been found [25]. During aging, a fast-to-slow transition in myosin isoform occurs, with a decrease in the abundance of type IIb MyHC and motor unit fibers. Furthermore, type IIx MyHC isoform is found in areas populated by fibers with type IIb MyHC isoforms in the skeletal muscle of young mice [26]. Finally, a greater percentage of skeletal muscle fibers of old rats show mixed populations of MyHC isoforms in the motor units compared with young rodents [27]. This heterogeneity may be secondary to age-related reinnervation that could not lead to MyHC transition within the motor unit.

Among the factors contributing to such age-associated reorganization of motor units is the reduction in number and function of α-motor neurons (i.e., decrease rate of axonal transport). When happening in the large motor neurons of fast-twitch motor units, an impaired reinnervation leads to loss of fiber type transition and atrophy [28]. Multiple factors contribute to the age-related loss of α-motor neurons, including genotoxic stress, impaired repair systems, and accumulation of DNA damage [29]. This is in line with findings in progeroid mice with deficient DNA repair showing DNA damage accrual and neuromuscular derangements phenotypically similar to those observed in old mice with impaired motor neurons and disrupted connectivity of the neuromuscular system [29]. Oxidative stress and nonenzymatic substrate glycosylation are additional contributing factors to neuromuscular derangements. Spinal motor neurons are particularly vulnerable to intermediate products of glycation that exert neurotoxic effects and trigger apoptotic cell death [30]. A defective antioxidant glutathione system further promotes the generation of glycated proteins during aging, thereby favoring the progressive demise of spinal motor neurons [30].

Changes in neuromuscular junctions (NMJs), the structures encompassing the presynaptic nerve terminal, intrasynaptic space, and post-synaptic muscle fibers in charge of transmitting action potentials from a nerve to the muscle [31], have also been implicated in muscle aging [32]. In particular, alterations in NMJ composition and function contribute to decreasing muscle mass and performance. Nonetheless, whether the age-related decline of muscle mass and strength is a cause or a consequence of NMJ changes remains to be established [33,34].

While changes in the morphology of the motor endplate and NMJ remodeling precede the loss of fast motor units during aging, reductions of the nerve terminal area and the number of muscle membrane post-synaptic folds have been reported. This corresponds to an impaired response of post-synaptic NMJ as slower motor nerve conduction velocity and reduced amplitude of muscle action potential occur [35].

Among the molecular determinants of age-associated NMJ alterations are the reduction in mitochondrial number and alterations in the organelle morphology (e.g., cristae disruption, hyperfused mitochondria) in axon terminals [36]. These ultrastructural changes are paralleled by oxidative damage and the reduced release of synaptic vesicles during pre-synaptic nerve terminal depolarization with muscle fiber denervation and atrophy occurring in a fiber-dependent manner [37,38]. Among the proposed molecular mechanisms of age-related reduced muscle mass and strength are the progressive inability of motor neurons to re-innervate denervated or regenerating muscle fibers, impaired coupling of excitation–contraction, and reduced proliferation of MSCs [39,40].

MSCs, located between the basal lamina and the sarcoplasma, are a dedicated pool of stem cells participating in muscle mass maintenance in the steady state and favoring regeneration upon muscle injury [41,42]. Changes in MSC number and function have been described during aging, with negative effects on muscle homeostasis and regeneration that can ultimately contribute to muscle dysfunction and accelerate the progression of sarcopenia [43,44,45]. MSCs exist and function in a rich microenvironment of extracellular matrix proteins and a plethora of cells (i.e., capillary endothelial cells, fibrocyte/adipocyte progenitors, and immune cells) [46,47,48,49] and secreted factors that are collectively referred to as MSC niche [50]. This rich and complex set of signals supports muscle regeneration through its stages by regulating MSC quiescence during muscle homeostasis and their activation in the setting of an injury. Upon completion of muscle regeneration, the MSC niche signals the return to quiescence and maintenance of the MSC pool [51,52]. Age-associated changes of the MSC niche have been shown to trigger MSC activation, differentiation, and senescence despite MSC quiescence and preservation [43,53]. MSCs maintain their regenerative potential during long periods of quiescence by enacting mitophagy- and autophagy-mediated recycling that preserves cell viability [54,55,56]. With advancing age, these processes become inefficient and MSC senescence occurs [57,58]. In this setting, the aged muscle niche signals the replacement of muscle tissue with fibrotic and adipose tissue [59]. However, exposing old MSCs to a young niche is not sufficient to avoid MSC senescence, indicating that intrinsic and extrinsic factors/defects trigger age-related muscle wasting [58].

## 3. Age-Related Muscle Changes: An Immunological Perspective

Immune cells are a critical component of the MSC niche and are intricately linked to the maintenance of muscle homeostasis and efficient regeneration following an injury [60]. The response of muscle to an acute injury is an excellent example of how crosstalk between MSCs and resident and recruited immune cells orchestrates tissue repair. This phenomenon is characterized by two temporally distinct phases, a proinflammatory and anti-inflammatory phase, and involves the coordinated and finely tuned activation of both resident and newly recruited immune cells [61,62,63]. The initial proinflammatory phase is marked by the release of proinflammatory cytokines (i.e., interleukin (IL)-1b, IL-6, tumor necrosis factor-α (TNF-α), interferon-γ (IFN-γ)) and chemokines (i.e., monocyte chemoattractant protein 1) from damaged muscle fibers and activated resident macrophages, followed by the activation of quiescent MSCs and recruitment of immune cells [62]. Once recruited, immune cells such as neutrophils, monocytes, and T cells amplify the inflammatory response and guide tissue regeneration. Neutrophils are among the first innate immune cells to be recruited from the circulation, and their main task is to clear the damaged site of cellular debris and prepare the scene for the arrival of inflammatory monocytes [64]. Macrophages are the most abundant innate immune cell in both the steady state and in the setting of muscle injury and are indispensable for all stages of myogenesis. The proinflammatory milieu drives inflammatory monocyte differentiation into inflammatory macrophages (or M1), which in turn produce inflammatory cytokines that stimulate MSC proliferation and expansion [49,65,66]. As muscle repair proceeds, M1 macrophages differentiate into anti-inflammatory cells generating M2 macrophages that stimulate myocyte differentiation and fusion [49,65]. Concomitantly, regulatory T cells are engaged and, together with M2 macrophages, drive myogenesis through the production of IL-10 [65,67,68].

The impaired ability of muscles to regenerate during aging has also been attributed to quantitative and qualitative changes of the MSC pool [69]. Furthermore, studies have highlighted a critical contribution of age-related changes in the number and function of immune cells, especially macrophages, to the decline of the regenerative capacity of muscles [43,70]. Indeed, aging is accompanied by changes in the levels of cytokines and growth factors produced by macrophages and necessary for adequate muscle regeneration. Old macrophages produce less growth differentiation factor 3 [71] and more osteopontin [72], thus resulting in impaired muscle regeneration following an injury. Age-related changes in myeloid cells can also affect muscle homeostasis and the outcome of muscle regeneration through a dysregulated M1/M2 macrophage shift [73,74], as well as alterations in their phagocytosis and autophagy properties [75]. Aged muscles are characterized by a greater number of M2 macrophages in response to an injury as well as in the steady state, despite the predominance of pro-inflammatory cytokines in both the muscle and the circulation as part of a systemic inflammation associated with aging [70]. However, although the levels of proinflammatory cytokines such as TNF-α [76] and IL-1b [77] are increased in both uninjured and injured aged muscles, the expression of the proinflammatory mediator IFN-γ is reduced [78]. On the other hand, levels of IL-10, a critical cytokine that regulates the switch from M1 to M2 macrophages in the aged muscle following an injury are much higher compared with young muscles [59]. It is plausible that this dysregulated cytokine environment in the aged muscle contributes to a dysregulated M1/M2 macrophage shift. Moreover, senescence-associated changes occurring in muscle resident mononuclear cells, including endothelial cells, fibro adipogenic progenitors (FAPs), and macrophages, might also alter the injury response via a greater production of the monocyte-recruiting chemokine CCL2 and the anti-inflammatory mediator IL-10 [78].

Therefore, the question arises as to why a biased increase in M2 macrophages can compromise the regeneration of aged muscle. As previously mentioned, it may be hypothesized that even a subtle imbalance in the ratio between M1 and M2 macrophages might adversely affect myogenesis [49,66].

Of note, not all age-associated changes in macrophage function and phenotype are attributable to the muscle microenvironment. When human myoblasts are incubated with conditioned media obtained from monocyte-derived macrophages isolated from the blood of old individuals, proliferation was significantly reduced compared to conditioned media derived from macrophages of young individuals [79]. These findings suggest that age-associated changes in macrophages occur, at least partly, independent of the aged muscle environment, at the level of the bone marrow [79].

In addition to the role of macrophages, a successful muscle regeneration depends on the presence of both cytotoxic T lymphocytes (CTLs) and regulatory T (Treg) cells, the depletion of either of which slows regeneration [60]. Aging has been linked to defects in the recruitment or function of both Treg cells or CTLs, thereby slowing the regeneration of aged muscle following injury [80]. This defect seems to be due to a reduction in the levels of IL-33 in old muscle, a cytokine necessary for Treg cell recruitment [80].

Optimal myogenesis depends on crosstalk between CTLs, Treg cells, and macrophages to regulate the action of IFN-y, a CTL-produced cytokine that is critical for timely M1 to M2 shift. Perturbations of this crosstalk during aging have been associated with impaired regeneration [78,81].

A normal muscle regeneration following an injury depends on the timely shift from a proinflammatory to an anti-inflammatory tissue environment that supports early activation and proliferation of MSCs. The latter events are facilitated by proinflammatory cytokines produced by M1 macrophages, while later myoblast fusion and myofiber growth are supported by IL-10 produced by M2 macrophages and Treg cells. In mouse models of muscle injury, the muscle is fully regenerated within 30 days of injury and inflammation is completely resolved, with the number of intramuscular immune cells back to basal levels [60]. However, the aged muscle regenerates slower, and the process is characterized by increased fibrosis and fat accumulation due to the predominance of IL-10-producing M2 macrophages and senescent FAPs [70,78].

Given the close relationship between immune cell senescence and suboptimal muscle regeneration during aging, an ideal therapeutic strategy would be one that can exert a positive effect on aged immune cells and the muscle microenvironment. Interestingly, both the immune system and the muscle are positively modulated by exercise in older adults. Indeed, studies have demonstrated a positive effect of exercise on the ability of aged muscle to regenerate [82,83,84]. One way whereby exercise might exert this beneficial effect is through a direct or indirect effect on the immune system. In aged muscle, exercise has been shown to increase the level of both pro- and anti-inflammatory cytokines, which are indispensable to adequate the MSC response to injury [85]. Likewise, exercise is associated with increased levels of leukocyte derived pro-regenerative cytokines [85].

Another possible way through which exercise might improve muscle performance during aging is through the induction of trained immunity. This is a biological process induced in innate immune cells, such as inflammatory monocytes, following exposure to certain pathogens, inflammatory cytokines, and self-derived damage associated with molecular patterns (DAMPs) typically released upon tissue injury [86]. A hallmark of trained immunity in monocytes is a hyperresponsive functional state, whereby a secondary exposure to the same or a different stimulus results in increased cytokine production by trained monocytes [86]. The trained immunity phenotype is dependent on mTOR activation and relies on epigenetic and metabolic reprogramming at the level of myeloid progenitors in the bone marrow and the spleen, giving rise to hyperresponsive monocytes [87]. This process has also been described in tissue-resident non-immune cells such as stem cells. Although little is known about this process in the context of muscle injury, a recent study showed that injury-experienced MSCs repaired injured muscle faster and more efficiently following a secondary injury compared with injury-naïve MSCs [88]. Moreover, authors found an increased level of myeloid-derived regeneration-associated cytokines in muscle following a second injury, suggesting that trained immunity is also induced in monocytes during muscle injury [88]. Additional studies are needed to dissect the mechanisms by which muscle injury leads to the induction of trained immunity. Considering that exercise based on eccentric muscle contractions is frequently associated with muscle (micro)damage [89], it will be interesting to examine whether certain types of exercise might induce trained immunity, which could improve muscle performance via direct action on MSCs and the innate arm of the immune response in older adults.

While macrophages have been attributed specific roles in skeletal muscle repair and regeneration, little is known about the contribution of resident macrophages to non-injured skeletal muscle metabolism and adaptation, as well extracellular matrix remodeling under external stimuli (e.g., exercise). This gap in knowledge is mostly due to limited methodological approaches for macrophage characterization. However, Kosmac et al. [90], using an immunohistochemistry protocol validated by flow cytometry, have shown that a mixed (M1/M2 phenotype) population of macrophages expressing specific markers (i.e., CD11b, CD14, CD68, CD86 and CD206) exists in non-injured human skeletal muscle. A smaller population of CD11b+/CD206− macrophages were also identified in resting skeletal muscle, and variations in the relative abundance of this macrophage population were proposed to reflect pivotal changes in the skeletal muscle microenvironment [90]. Indeed, an increase in skeletal muscle macrophages has been observed in response to endurance exercise training (e.g., cycling) [91]. Following endurance exercise training, changes in M2 macrophages were observed and associated with variations in the levels of insulin growth factor, a promoter of muscle growth, and genes involved in extracellular matrix remodeling. Endurance exercise training was also associated with a reduction in the levels of mediators of inflammation (i.e., IL-6) and muscle atrophy (i.e., muscle-specific RING finger protein 1) [91]. Notably, mechanical loading was identified as a trigger of skeletal muscle cells and macrophage crosstalk via the release of matrix metalloproteinase 14 for extracellular matrix remodeling [92]. Additional studies are warranted to understand the molecular mechanisms eliciting trained immunity for the improvement of muscle performance.

## 4. Age-Related Changes of Mitochondria in the Muscle

Mitochondria are crucial for carrying out a set of processes other than energy production in the cell, including calcium and iron buffering, amino acid and lipid metabolism, thermogenesis, apoptosis, and reactive oxygen species (ROS) signaling [93,94,95]. Age-related biochemical and bioenergetic changes, including reduction of mitochondrial volume and oxidative capacity and production of high levels of ROS, have been observed and associated with perturbations in mitochondrial dynamics, biogenesis, and apoptosis [93,94,95].

Although such changes have been described in several tissues during aging, the skeletal muscle shows specific vulnerability to mitochondrial dysfunction owing to its high reliance on oxidative metabolism. For instance, mitochondria in motor nerve terminals are necessary not only to provide enough ATP to assist excitation–contraction coupling but also to buffer calcium load [96]. Similar to the muscle, the brain, a high-energy-demanding and post-mitotic organ, is especially sensitive to mitochondrial dysfunction and ensuing oxidative stress [97]. Indeed, the inactivation of mitochondrial complex I in Parkinson’s disease likely fuels the production of peroxide anion and peroxynitrite with consequent lipid peroxidation and amine–aldehyde adducts formation. As a compensatory response, mitochondrial nitric oxide synthase activity is triggered, and mitochondrial biogenesis is stimulated [97].

Sarcopenic rats with deranged NMJs show a reduced expression of genes implicated in mitochondrial energy metabolism compared with their wildtype counterparts [98]. A low ATP production and impaired calcium buffering in subsarcolemmal mitochondria near NMJs may impinge on both neurotransmission and vesicular recycling [99].

**Figure 2:**
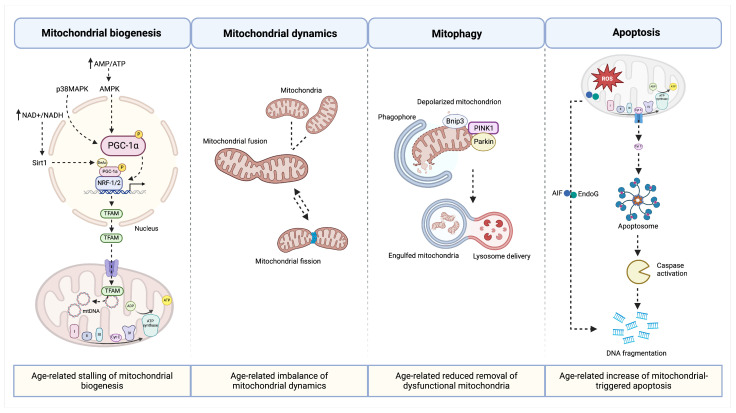
Schematic representation of pathways implicated in reduced mitochondrial quality and muscle aging. Created with https://www.biorender.com/ (accessed on 24 March 2024).

Age-related reductions of mitochondrial function have also been described in human skeletal muscle [100] and associated with declines in muscle strength and walking performance [101,102]. Although the molecular mechanisms of such detrimental organelle changes are not completely deciphered, the accumulation of dysfunctional mitochondria due to an impaired mitophagy has been reported [103]. A stalling of mitochondrial biogenesis has also been documented during muscle aging [104]. However, mitochondrial biogenesis, the self-renewal of mitochondria from existing organelles, triggered in the setting of energy-demanding conditions (e.g., developmental signals, environmental stressors) [105] is in a balance with the removal of damaged organelles [106,107]. The hypothesis of a coordinated molecular machinery for preserving mitochondrial homeostasis in a process of mitochondrial quality control (MQC) that culminates into myocyte homeostasis has been hypothesized [9,108]. The accrual of deranged mitochondria with aging, together with mitochondrial energy constraints due to long-standing impaired function of the electron transport chain, favors this hypothesis and poses a clear limitation to the cell’s capacity of executing the energetically expensive mitophagy process [103]. However, whether the failure of a coordinated MQC is a culprit or a bystander of muscle aging and the development of sarcopenia is yet to be established (Figure 2).

## 5. Mitochondrial Delivery as a Remedy in Muscle Regeneration

In the view of mitochondrial dysfunction being a driver of muscular dyshomeostasis and functional decline, mitochondrial transplantation has recently been proposed as a therapeutic strategy for muscle bioenergetic reprogramming and recovery similar to what was shown for the treatment of diseases caused by defective mitochondria [109]. The rationale underlying this strategy lies in the observation that transplanted mitochondria can support energy production and ATP storage and can also rescue cellular function by replacing damaged mitochondrial DNA (mtDNA) [110,111,112].

Studies in preclinical models of ischemia–reperfusion have shown that autologous transplanted mitochondria in the ischemic heart are able to support myocardial cell viability and recovery from ischemia–reperfusion injury [16]. Subsequent investigations in rabbits confirmed such findings showing that the delivery of autologous bioenergetically-competent mitochondria into ischemic heart areas during early reperfusion reduced infarct size and preserved myocardial cell bioenergetics and viability [18,19]. While the success of mitochondrial transplantation in the setting of cardiac ischemia–reperfusion has been widely investigated, more recent findings show that the transplantation of mitochondria ameliorates hindlimb function in preclinical models of acute limb ischemia [113]. Indeed, the transfer of autologous mitochondria isolated from the right rectus muscle to an ischemic region shortly before reperfusion induced a partial restoration of muscle function in mice [114]. Recent studies have also indicated that mitochondrial transplantation reduces ischemia–reperfusion injury following cardiac arrest and modulates immune responses via a synergistic effect with other therapeutic remedies (e.g., hypothermia) [115]. Finally, transplanting intermyofibrillar mitochondria from mouse skeletal muscle to myoblasts has also been shown to induce an improvement in myoblast bioenergetics [112]. The transplantation of murine muscle mitochondria into human fibroblasts holding mtDNA mutations has also been shown to improve mitochondrial dynamics, metabolism, and redox homeostasis [112]. The mitochondrial transfer occurred via extracellular vesicles (EVs), gap junctions, micropinocytosis, and tunneling nanotubes, thereby paving the way to less invasive techniques for organelle transplantation that exploits EV trafficking [112]. The systemic delivery of mitochondria in murine models of BaCl_2_-injured muscles and dexamethasone-induced muscle atrophy has been shown to improve muscle regeneration and restore muscle function [23,116].

These preclinical studies served as a reference point for developing clinical investigations. Autologous mitochondrial transplantation has been performed in a trial involving pediatric patients with ischemia–reperfusion injury to replenish damaged mitochondria with organelles isolated from non-ischemic skeletal muscle via intramyocardial delivery [20]. Finally, the incorporation of mitochondria into mesenchymal stem cells following organelle transplantation has been shown to improve repair of arterial lung and cardiac tissue [21,22].

Recently, mitochondrial transplantation has also proved beneficial in the setting of lower limb ischemia–reperfusion injury with molecular mechanisms that are presently unclear [117]. As a preliminary indication, mitochondria derived by human umbilical cord mesenchymal stem cells (hMSCs) taken up by skeletal muscle cells and adipocytes were able to induce an increase in the content of the mitochondrial fusion protein, optic atrophy 1 (OPA1), and uncoupling protein 1 [117]. As an effect of such changes, skeletal muscle mitochondrial function was rescued, and adipocyte browning was promoted. Furthermore, a reduction in cellular apoptosis was observed with an increase in muscle tissue repair and motor function recovery [117].

Although the evidence on the beneficial effects of mitochondrial transplantation has increased since the first investigations on the topic, whether this strategy could serve as a remedy against age-related sarcopenia remains to be established. As for the current knowledge, the transplantation of non-autologous mitochondria has been shown to improve mitochondrial bioenergetics with short-term duration [17] and has therefore been indicated as a strategy that may be more suitable in the setting of acute stress.

## 6. Conclusions

Mitochondrial transplantation and the restoration of the pool of MSCs have been indicated as pivotal factors for achieving skeletal muscle homeostasis and regeneration. There is evidence of a tight coordination between intrinsic (skeletal muscle molecular and ultrastructural variations) and extrinsic (mononucleated MSCs) signaling pathways that may culminate into the modulation of mitochondrial biogenesis and remodeling for ensuring functional organelles in the muscle. The delivery of mitochondria in the site of damaged tissues as well their inclusion into MSCs is regarded as a novel approach to blunt age-related muscle wasting. This possibility warrants investigation through ad hoc designed studies.
